# Direct laser writing-enabled 3D printing strategies for microfluidic applications

**DOI:** 10.1039/d3lc00743j

**Published:** 2024-04-04

**Authors:** Olivia M. Young, Xin Xu, Sunandita Sarker, Ryan D. Sochol

**Affiliations:** a Department of Mechanical Engineering, University of Maryland College Park, 2147 Glenn L. Martin Hall College Park MD 20742 USA rsochol@umd.edu; b Maryland Robotics Center, University of Maryland College Park MD 20742 USA; c Institute for Systems Research, University of Maryland College Park MD 20742 USA; d Fischell Department of Bioengineering, University of Maryland College Park MD 20742 USA; e Robert E. Fischell Institute for Biomedical Devices, University of Maryland College Park MD 20742 USA; f Department of Mechanical and Industrial Engineering, University of Massachusetts Amherst MA 01003 USA

## Abstract

Over the past decade, additive manufacturing—or “three-dimensional (3D) printing”—has attracted increasing attention in the *Lab on a Chip* community as a pathway to achieve sophisticated system architectures that are difficult or infeasible to fabricate *via* conventional means. One particularly promising 3D manufacturing technology is “direct laser writing (DLW)”, which leverages two-photon (or multi-photon) polymerization (2PP) phenomena to enable high geometric versatility, print speeds, and precision at length scales down to the 100 nm range. Although researchers have demonstrated the potential of using DLW for microfluidic applications ranging from organ on a chip and drug delivery to micro/nanoparticle processing and soft microrobotics, such scenarios present unique challenges for DLW. Specifically, microfluidic systems typically require macro-to-micro fluidic interfaces (*e.g.*, inlet and outlet ports) to facilitate fluidic loading, control, and retrieval operations; however, DLW-based 3D printing relies on a micron-to-submicron-sized 2PP volume element (*i.e.*, “voxel”) that is poorly suited for manufacturing these larger-scale fluidic interfaces. In this *Tutorial Review*, we highlight and discuss the four most prominent strategies that researchers have developed to circumvent this trade-off and realize macro-to-micro interfaces for DLW-enabled microfluidic components and systems. In addition, we consider the possibility that—with the advent of next-generation commercial DLW printers equipped with new dynamic voxel tuning, print field, and laser power capabilities—the overall utility of DLW strategies for *Lab on a Chip* fields may soon expand dramatically.

## Abstract 2

This Tutorial Review highlights strategies for leveraging the micron-to-submicron-scale additive manufacturing technique, “direct laser writing”, to enable 3D microfluidic technologies.

## Graphical abstract

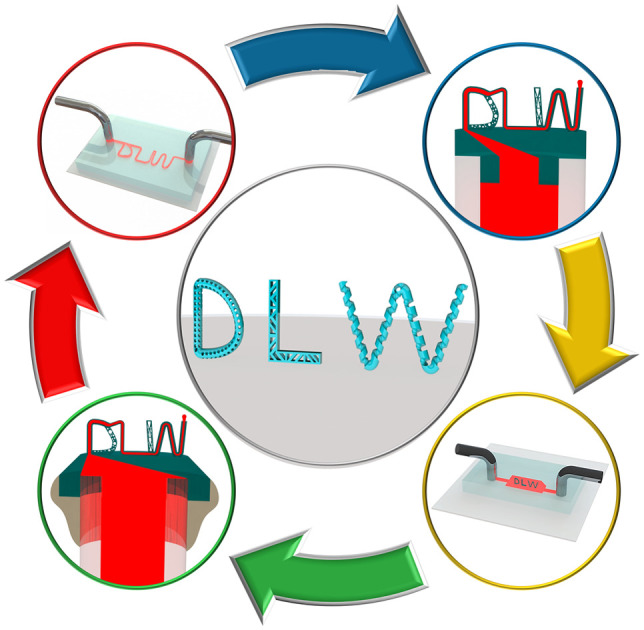

## 1. Introduction

**Fig. 1:**
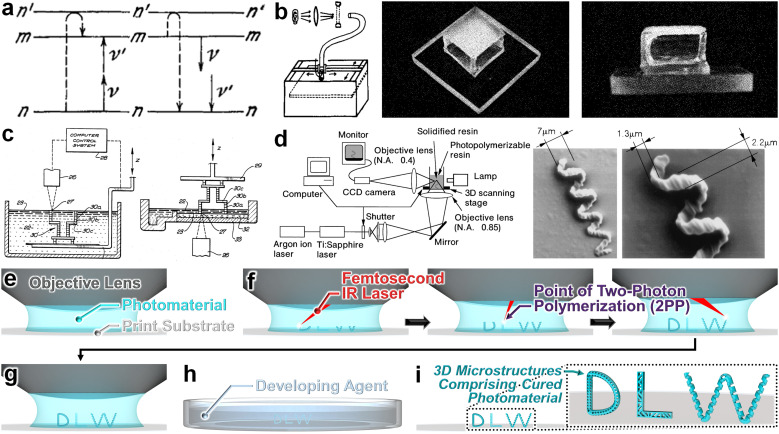
Fundamental concepts and developments for “direct laser writing (DLW)”. (a) Theory of two-photon absorption. Dashed lines = behaviour of an atom; upward and downward arrows represent absorbed and emitted photons, respectively.^1,2^ (b) Conceptual illustration and experimental results from the first demonstration in the literature for fabricating a three-dimensional (3D) model by exposing a liquid photomaterial to UV light in a point-by-point, layer-by-layer manner.^6^ (c) Conceptual illustrations of two configurations of the “stereolithography apparatus (SLA)” for building 3D objects *via* point-by-point, layer-by-layer polymerization of a photomaterial.^7^ (d) Schematic of the optical system and experimental results from the first demonstration in the literature for fabricating 3D microstructures *via* two-photon polymerization (2PP).^8^ (e–i) Conceptual illustrations of a representative DLW manufacturing process to produce 3D “DLW” microstructures. (e) Uncured photomaterial atop a print substrate. (f) A pulsed infrared (IR) laser is scanned point by point and/or layer by layer to initiate 2PP in target locations. (g) Completion of the DLW printing process. (h) The print is immersed in a developing agent to remove any uncured photomaterial. (i) Completion of the development process results in final microstructures—still adhered to the print substrate—comprising cured (crosslinked) photomaterial.

Nearly a century ago, future Nobel Laureate Maria Goeppert Mayer theorized the concept of two-photon absorption in her doctoral thesis (Fig. 1a).^1,2^ Three decades later, following the invention of the pulsed ruby laser by Theodore Maiman,^3^ Kaiser and Garrett reported the first experimental demonstration of Goeppert Mayer's theory *via* two-photon excitation in CaF_2_:Eu^2+^.^4^ Then, after another three decades, Denk *et al.* extended this concept to achieve two-photon excitation microscopy.^5^ Meanwhile, in a different area of research, Hideo Kodama reported several strategies for building three-dimensional (3D) objects by exposing liquid-phase photohardening polymers to light (Fig. 1b).^6^ Soon after, Charles (Chuck) Hull patented a similar concept of a “stereolithography apparatus (SLA)”, which entails using a vat of liquid-phase photomaterial and ultraviolet (UV) light to photocure (*i.e.*, crosslink) the material in a point-by-point, layer-by-layer routine (Fig. 1c).^7^ Notably, in 1997, Maruo *et al.* combined these two concepts of two-photon absorption and photopolymerization-based additive manufacturing to build fully 3D microstructures by means of two-photon polymerization (2PP) phenomena (Fig. 1d).^8^ This 3D manufacturing strategy has since come to be referred to as “laser lithography” and, more commonly, “direct laser writing (DLW)”. A decade later, Nanoscribe GmbH—a scientific spin-off from the Martin Wegener group at the Karlsruhe Institute of Technology—was founded to commercialize microfabrication tools for 3D printing with submicron resolution,^9^ which has played a key role in the international adoption of DLW in both research and industrial settings.

The DLW fabrication process consists of three primary steps. First, a photosensitive material (*e.g.*, a negative-tone photoresist) is typically deposited onto a print substrate and loaded into a DLW printer (Fig. 1e). It is important to note that researchers have demonstrated a vast array of photomaterials as well as photocomposites to be compatible with 2PP-based fabrication processes, as discussed in numerous recent review articles.^10–14^ Briefly, to be compatible with DLW protocols, the components of the photomaterial—*i.e.*, the photoinitiator(s), monomer(s), and/or oligomer(s)—must be transparent in the wavelength (*λ*) of the laser used for DLW, the monomer/oligomer must be transparent in the two-photon absorption wavelength (*λ*/2), and the photoinitiator must: (i) absorb at the two-photon absorption wavelength, (ii) have a high cross section (*i.e.*, more likely to transition to a higher state when sufficient energy is absorbed), and (iii) have a high quantum efficiency (*i.e.*, probability the molecule will break down into active fragments).^14–16^ Acrylate-based photoresists represent one of the most prominent classes of photomaterials for DLW with benefits including low costs, high availability, transparency at visible and near-infrared wavelengths, stability after polymerization, and ease of development.^17–21^ In addition, researchers have reported a number of other material systems for use in DLW protocols, such as epoxy-based photoresists (*e.g.*, SU-8),^22–24^ hydrogels including poly(ethylene glycol) diacrylate (PEG-DA) and gelatin methacryloyl (Gel-MA),^25–28^ organic/inorganic hybrids,^29–31^ and modified photoresins.^32–34^

To print desired structures, the second step involves using scanning a tightly focused femtosecond infrared (IR) laser in a point-by-point and/or layer-by-layer routine to initiate spatially controlled 2PP (or multi-photon polymerization) of the photomaterial at designated locations in 3D space (Fig. 1f).^35–38^ Lastly, following completion of the 2PP printing process (Fig. 1g), development procedures are implemented with regard to the type of photomaterial and/or print substrate employed.^39–41^ Generally, the assembly comprising the print substrate and the polymerized object(s) is removed from the printer and immersed in a developing agent (Fig. 1h), such as propylene glycol methyl ether acetate (PGMEA), which is often followed by secondary cleaning steps with solvents such as isopropyl alcohol (IPA).^42–44^ Such development processes are needed to ensure that any residual (uncured) photomaterial has been removed.^45–47^ Ultimately, these DLW fabrication processes culminate in the production of 3D micro/nanostructured entities comprising cured photomaterial (Fig. 1i).

The reliance on 2PP affords three distinguishing advantages for DLW compared to alternative additive manufacturing approaches. The first key benefit is the scale at which 3D structures can be fabricated, with researchers demonstrating feature resolutions on the order of 100 nm.^48–51^ Historically, manufacturing systems at such length scales has relied predominantly on clean room-based microfabrication approaches (*e.g.*, photolithography, e-beam lithography) that are typically limited by intrinsic geometric constraints.^52–54^ DLW, thus, offers distinctive utility as one of the only pathways for high 3D design control at micron-to-submicron scales.^55–57^ A second advantage is the ability to spatially localize polymerization reactions to the singular point of 2PP—*i.e.*, the photocured volume element or “voxel”—thereby preventing undesired photopolymerization along the laser path, as is the case for SLA and similar “vat photopolymerization (VPP)” methods.^58–61^ As a result, DLW bypasses the flat build surface restriction inherent to most VPP techniques to allow structures to be printed in a wide variety of scenarios, such as directly onto curvilinear surfaces or alternative, non-standard substrates.^62,63^ This capability also facilitates DLW strategies that overcome the near-ubiquitous single-print-material limitation of VPP approaches to enable multi-material 3D printing.^64–68^ A third advantage is the speed with which micro/nanostructured 3D objects can be produced. In contrast to extrusion-based additive manufacturing techniques—*e.g.*, fused deposition modelling (FDM) or fused filament fabrication (FFF) and direct ink writing (DIW)—which suffer from an inherent trade-off between minimum feature size (based primarily on the nozzle diameter) and total print time,^69–72^ such constraints can be circumvented for DLW. Akin to SLA, the use of galvanometric micromirrors for DLW allows for the voxel to be scanned rapidly to designated positions,^73–76^ facilitating DLW-based print speeds (*i.e.*, laser scanning speeds) ranging from 100 mm s^−1^ to those in excess of 1000 mm s^−1^ without compromising the micron-to-submicron-scale resolution.^77,78^

In combination, these advantages have enabled researchers to achieve new classes of 3D micro/nanostructured systems for both fundamental and applied research across fields ranging from the life sciences,^46,79,80^ materials science,^81–84^ and metamaterials^85–87^ to MEMS,^88–90^ microrobotics,^91–94^ optics,^95–97^ and photonics.^98–100^ For the *Lab on a Chip* community, however, the foundational role of microfluidic devices brings about distinctive obstacles to the use of DLW, which notably stem from one of the most fundamental requirements for microfluidic technologies: macro-to-micro fluidic interfaces. It is nearly ubiquitous for microfluidic devices to rely on the use of macro-to-micro fluidic interfaces, such as inlet and outlet ports to support microfluidic loading and retrieval operations, respectively, as well as control ports that allow for off-chip regulation of on-chip processes.^101–106^ Unfortunately, the ability to facilitate such macro-to-micro fluidic interfaces represents the primary hurdle to the efficacy of DLW for microfluidic applications. Specifically, this barrier arises from the micron-to-submicron size of the printing voxel, which while beneficial for resolving miniaturised structures, is poorly suited for point-by-point fabrication of millimetre-scale macro-to-micro fluidic interfaces. Consequently, researchers have devised and investigated a wide range of strategies to circumvent this inherent limitation of DLW in an effort to facilitate its use as an enabling technology for microfluidic applications, which we review herein.

## 2. Master mould fabrication for microreplication *via* direct laser writing (DLW)

**Fig. 2:**
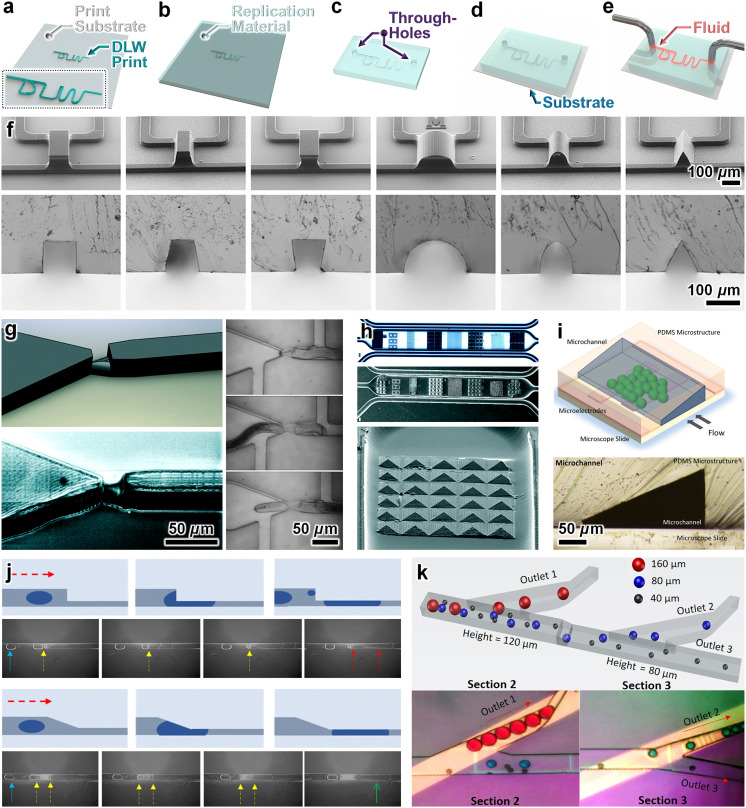
Microfluidic system fabrication based on the use of DLW-printed moulds for microreplication. (a–e) Conceptual illustrations of a representative fabrication protocol. (a) Master mould with DLW-printed microchannel structures. (b) Micromoulding of a material, such as polydimethylsiloxane (PDMS). (c) Following removal of the micromoulded material from the master mould, through-holes are produced at desired locations for macro-to-micro fluidic interfaces (*e.g.*, inlet/outlet ports). (d) The micromoulded material is enclosed using a substrate (*e.g.*, by bonding PDMS to glass). (e) Fluid is loaded into the enclosed microfluidic channel. (f–k) Representative examples in the literature. (f) Micrographs of (top) master moulds, and (bottom) corresponding replicated PDMS cross-sectional profiles for various microchannel designs.^124^ (g) Microchannel with geometry designed to trap plant parasitic nematodes.^127^ (h) Microfluidic channels with microtopographical features for studies of cell phisicobiology.^128^ (i) Sloped microfluidic channels for applying lateral spatial shear stress gradients to cells.^129^ (j and k) Microfluidic channels designed with gradual changes in height to enhance microdroplet: (j) stability,^130^ and (k) manipulations (*e.g.*, size-based sorting).^132^

Microreplication protocols—*e.g.*, “soft lithography” using polydimethylsiloxane (PDMS)—represent one of the most pervasive approaches for manufacturing microfluidic channels in research settings.^107–109^ Most such approaches, however, rely on the aforementioned conventional microfabrication techniques that are ill suited for applications that demand sophisticated microchannel geometries.^110^ As a result, the replicated microchannels and/or microstructures typically comprise “2.5D” geometries, such as rectangular microchannels and microfeatures with relatively straight sidewalls and a single, uniform height.^111–113^ Although there are pathways to achieve basic “quasi-2.5D” geometries (*e.g.*, microchannels with hemispherical cross sections, microchannels with different heights) *via* conventional means,^114–116^ DLW affords far more expansive geometric versatility in the production of master moulds for microreplication.^117–119^ Thus, one of the most straightforward routes to harness DLW for microfluidic applications is to use DLW to: (i) produce a master mould with features that would be difficult or infeasible to fabricate through standard microfabrication techniques (Fig. 2a), (ii) use the mould for microchannel replication (*e.g.*, with PDMS) (Fig. 2b), (iii) remove material (*e.g.*, punch/drill holes) at locations for the macro-to-micro fluidic interfaces (Fig. 2c), and then (iv) bond the microchannel to enclose the device (Fig. 2d) and enable microfluidic loading and retrieval operations (Fig. 2e).

DLW enables high customization in the design of master mould geometric characteristics,^120–123^ not only with regard to the cross-sectional profiles, but also to achieve microchannels and microfeatures with numerous and/or gradually tapered heights (Fig. 2f).^124–126^ Researchers have harnessed this geometric control to produce master moulds that support studies in the life sciences, such as to resolve tapered semi-cylindrical trapping channels for plant parasitic nematodes (Fig. 2g)^127^ as well as microfluidic devices with topographical features (*e.g.*, pyramid arrays) for investigations of cell physicobiology (Fig. 2h).^128^ One of the most prominent uses of DLW for master mould fabrication is to achieve microchannels with sloped cross-sectional profiles. For example, Soffe *et al.* created sloped microfluidic channels to achieve unique lateral spatial shear stress gradients for studies of cell mechanobiology (Fig. 2i).^129^ The Han group in particular has leveraged DLW for master mould production to improve on-chip microdroplet handling, such as to enhance droplet stability (Fig. 2j),^130^ pairing and merging,^131^ and critical manipulation functions (Fig. 2k).^132^

Although these works highlight the utility of DLW for 3D printing master moulds that can be integrated into standard microreplication protocols for fabricating microfluidic devices,^23,133^ there are two inherent limitations of such approaches. From a design perspective, a key restriction for DLW-printing moulds is that, despite allowing for more sophisticated quasi-2.5D geometries compared to their conventional microfabrication counterparts, they must still be mouldable to support effective microreplication. As a result, the use of DLW for printing microreplication master moulds is not appropriate for applications that require fully 3D microfluidic systems. The second limitation is associated with the current production capacity of DLW-based 3D printers. Specifically, conventional microfabrication methods are well established for fabricating microchannel master moulds over large areas (*e.g.*, onto standard 4′′ and 6′′ Si wafers) rapidly; however, point-by-point, layer-by-layer DLW is inefficient for such size scales, typically resulting in substantially longer fabrication times. Thus, the use of DLW for printing master moulds is better suited for cases in which smaller device footprints (*e.g.*, 1′′ wafers) are acceptable.

## 3. Enclosure of DLW-printed microstructures inside microfluidic devices

### 3.1. DLW of microstructures onto a planar substrate and then aligning and sealing a microchannel to enclose the system

**Fig. 3:**
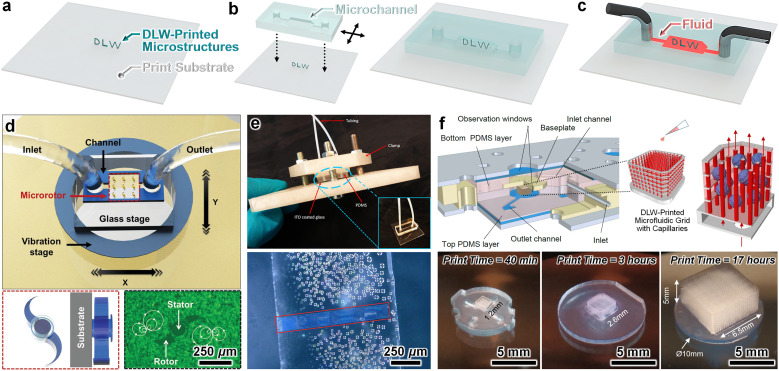
Microfluidic systems fabricated by enclosing DLW-printed 3D microstructures. (a–c) Conceptual illustrations of a representative fabrication protocol. (a) Microstructures DLW-printed onto a planar (*i.e.*, flat) substrate (*e.g.*, glass). (b) Channel enclosure. (Left) Alignment of an unenclosed microchannel with integrated macro-to-micro fluidic interfaces (*e.g.*, an unenclosed PDMS microchannel with inlet/outlet ports) to the microstructures; and then (right) enclosure of the microfluidic system (*e.g.*, *via* PDMS-to-glass bonding). (c) Loading of fluid into the enclosed microfluidic channel comprising DLW-printed 3D microstructures. (d–f) Representative examples in the literature. (d) PDMS-on-glass microfluidic system containing an array of DLW-printed 3D microrotors.^135^ (e) DLW-printed microstructures enclosed in microfluidic channels *via*: (top) mechanical clamping, and (bottom) PDMS-to-glass oxygen plasma bonding.^136^ (f) Microfluidic tissue culture system with DLW-printed 3D microfluidic capillary grids enclosed by mechanical clamping. (Top) Conceptual illustrations. (Bottom) Fabrication results.^137^

Given the numerous methods for enclosing microchannels developed by the *Lab on a Chip* community,^134^ one of the most facile strategies for fabricating a microfluidic device comprising DLW-printed microstructures entails: (i) DLW-printing the desired microstructure(s) onto a planar (*i.e.*, flat) substrate, such as a glass slide (Fig. 3a), and then (ii) aligning and sealing a microchannel—which includes the macro-to-micro fluidic interfaces—to enclose the microstructures (Fig. 3b), thereby supporting microfluidic operations (Fig. 3c). For example, Zhou *et al.* used DLW to print arrays of 3D microrotors onto an ITO-coated fused silica glass substrate and then aligned and plasma bonded a PDMS microchannel (fabricated *via* standard soft lithography protocols) to complete the microfluidic system (Fig. 3d).^135^ In an effort to study the effects of topographical features on cell and tissue behaviour associated with bone regeneration, Nouri-Goushki *et al.* demonstrated that in addition to microchannel plasma bonding approaches, mechanical clamp setups can be used to align and fluidically seal microchannels over DLW-printed microstructures (Fig. 3e).^136^ Recently, Grebenyuk *et al.* extended this mechanical clamping strategy to achieve a microfluidic perfusion system for large-scale engineered tissues that was founded on 3D microfluidic capillary grids fabricated using DLW (Fig. 3f).^137^

Although these strategies benefit from accessibility as they can be readily incorporated into traditional microfluidic device fabrication protocols, the critical limitation stems from fluidic sealing for the DLW-printed microstructures. Specifically, the considerable manufacturing challenges that would need to be addressed to not only match the geometry of the printed microstructures to that of the enclosing microchannel, but also align the microchannel to the microstructures precisely prior to sealing (*e.g.*, bonding or mechanical clamping) suggest that such strategies are not ideal for cases that require complete microfluidic sealing of the DLW-printed microstructures to all of the inner surfaces (*e.g.*, the sidewalls) of the microchannel. Thus, the use of these types of approaches is typically restricted to microfluidic applications for which the DLW-printed microstructures do not need to be microfluidically sealed to any surface of the enclosing microchannel.

### 3.2. DLW of microstructures inside an unenclosed microchannel and then sealing a planar substrate to enclose the system

**Fig. 4:**
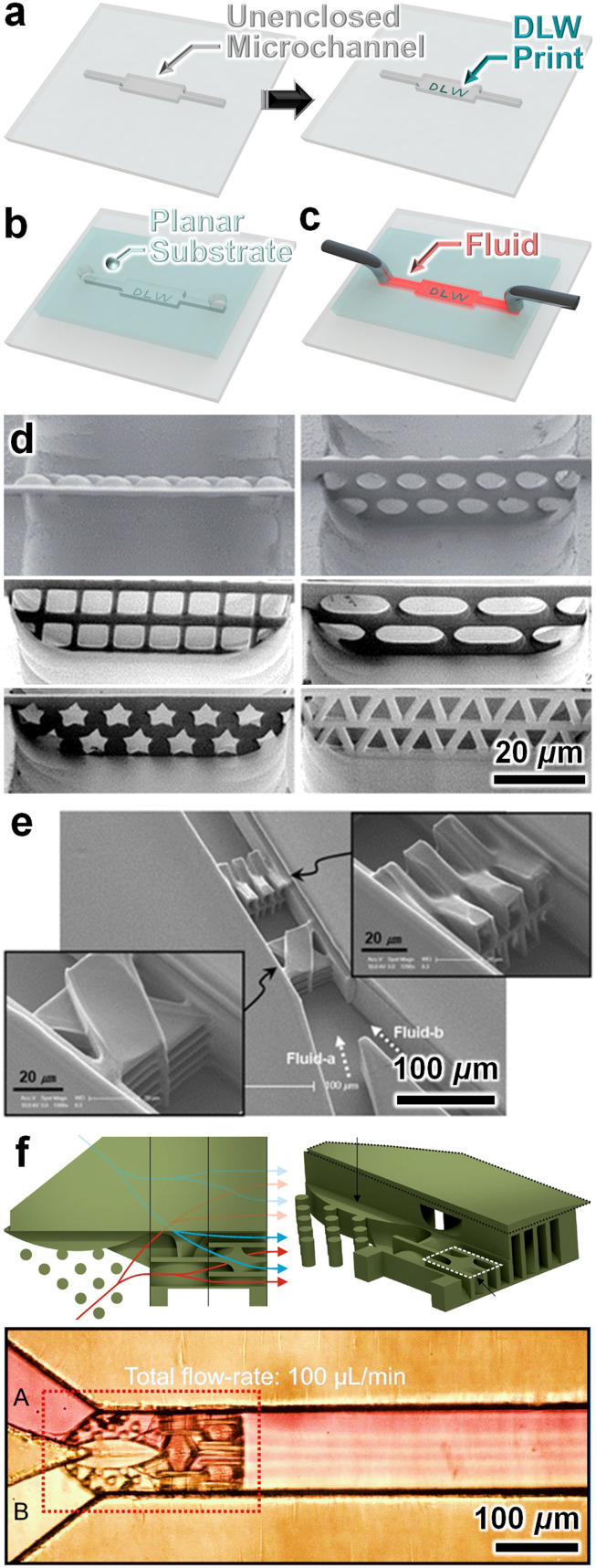
Microfluidic systems fabricated by DLW-printing 3D microstructures inside of an unenclosed channel and then enclosing the channel with a planar substrate. (a–c) Conceptual illustrations of an example fabrication protocol. (a) DLW-printing of microstructures directly inside of an unenclosed microchannel (*e.g.*, wet-etched glass). (b) Channel enclosure by sealing a planar substrate (*e.g.*, a PDMS slab) with integrated macro-to-micro fluidic interfaces atop the microchannel's open surface. (c) Loading of fluid into the enclosed microfluidic channel comprising DLW-printed 3D microstructures. (d–f) Representative examples in the literature. (d and e) Micrographs of microfluidic structures, including (d) microfilters with arbitrary pore designs^138^ and (e) multidirectional crossing manifold micromixers,^140^ which were DLW-printed inside wet-etched glass microchannels and then sealed using flat PDMS slabs.^138,140^ (f) A 3D micromixer that was DLW-printed inside unenclosed SU-8 photoresist-on-Si microchips and then sealed using a flat PDMS slab.^142^

For cases in which it is desired or required for DLW-printed microstructures to be microfluidically sealed to the microchannel sidewalls, a reverse strategy involves instead: (i) DLW-printing the desired microstructure(s) inside of an unenclosed microchannel (*e.g.*, wet-etched glass) (Fig. 4a), and then (ii) enclosing the microchannel with the DLW-printed microstructures by sealing a planar substrate (*e.g.*, bonding a flat sheet of PDMS that includes the macro-to-micro fluidic interfaces) onto the surface (Fig. 4b) to support microfluidic operations (Fig. 4c). The Sun group was one of the earliest pioneers of such approaches, reporting the use of standard wet etching protocols to fabricate open glass microchannels in which 3D microstructures, such as filters with arbitrary pore designs (Fig. 4d)^138^ and “overpass” microfluidic vias,^139^ were printed using DLW, and then enclosing the microfluidic systems using flat PDMS slabs with integrated inlet/outlet ports. Lim *et al.* reported a similar method of DLW-printing crossing manifold micromixers inside unenclosed SU-8 photoresist-on-glass microchannels, and then plasma bonding PDMS to enclose the microfluidic device (Fig. 4e).^140^ Other groups have furthered these strategies to demonstrate additional DLW-printed microfilters^141^ and 3D microfluidic mixers (Fig. 4f)^142^ with more complex geometries. To engineer cardiac tissues toward heart-on-a-chip microsystems, Jayne *et al.* reported an alternative approach in which they used DLW to print 3D cell scaffold structures (“microcages”) onto the sidewalls of an open PDMS channel and then bonded the system to a glass substrate to enclose the device.^143^

From a practical standpoint, one of the main drawbacks of such approaches stems from the methods used for microchannel fabrication. It can be difficult to achieve high sealing integrity for microstructures DLW-printed onto PDMS sidewalls,^124^ which accounts for the reported use of wet etching^138,139^ and laser ablation^141^ protocols to achieve glass microchannels that promote structure-to-channel adhesion. Such protocols, however, are not as accessible to many investigators in *Lab on a Chip* fields as those associated with standard photolithography. Yet, even for approaches that rely on DLW-printing structures onto SU-8 photoresist-based microchannels,^140,142^ there remain two key challenges. First, although alignment of the enclosing planar substrate is facile, arduous alignment protocols are needed prior to commencing the DLW printing process to ensure that the printed microstructures are oriented properly with respect to the microchannel. This alignment is critical not only along the lateral, longitudinal, and rotational axes of the channel, but also with respect to the channel depth as: (i) printing a microstructure that is too low in the channel will lead to considerable fluid flow that bypasses the structure(s), which can greatly diminish device efficacy (*e.g.*, particles bypassing a filter, fluids bypassing a mixer); or conversely, (ii) printing a microstructure that is too high in the channel can disrupt the ability to perform the final sealing step (*e.g.*, the device cannot be enclosed), leading to device failure. Because these alignment protocols are typically executed manually (*e.g.*, by hand/eye under brightfield microscopy),^144^ user skill can be a pivotal determinant in the degree of time and labour associated with executing such procedures, which can also negatively affect repeatability and reproducibility. The second challenge is that a wide range of factors, such as the aforementioned alignment requirements as well as both the surface roughness and material properties of the DLW-printed microstructures, present considerable obstacles to achieving a fluidic seal between the exposed surface of the DLW-printed structure(s) and the complimentary surface of the enclosing substrate. Thus, while these approaches are effective for microfluidic applications that necessitate structure-to-sidewall sealing, they are often not ideal for cases that rely on achieving complete fluidic sealing of the DLW-printed structure(s) to the entire luminal surface of the enclosed microchannel.

## 4. DLW of microstructures within fully enclosed microfluidic devices

### 4.1. DLW inside enclosed PDMS-on-glass microfluidic systems

Due to the widespread adoption of PDMS-on-glass microfluidic devices across the *Lab on a Chip* community,^145–147^ there has been considerable interest in the ability to use such systems as a backbone for DLW-printing microstructures for microfluidic applications. Conventional PDMS-on-glass systems, however, present a number of inherent barriers to DLW-printing of microstructures directly inside of the enclosed microchannels. Several of these obstacles stem from the use of PDMS as a microchannel material. For instance, many organic solvents that are employed in standard development protocols for DLW-based prints are incompatible with PDMS,^148,149^ which can lead to device degradation as such developers are perfused through the microchannels following the DLW 3D printing process. Although this issue can be circumvented through the use of different photoresist-developer material systems, the gas permeability of PDMS represents a more substantial hurdle. Specifically, this material characteristic gives rise to a thin layer of oxygen along the PDMS surface that can not only disrupt photopolymerization phenomena,^150–152^ but also lead to unintended print failures from burning and/or micro-explosions (*i.e.*, “bubbling”) of the photoresist while DLW printing is performed adjacent to the PDMS surface.^153–155^ Other obstacles stem from geometric factors associated with the microchannel cross-sectional profiles of standard PDMS-on-glass microfluidic devices. In particular, common soft lithography protocols produce microchannels with relatively straight channel sidewalls (*e.g.*, <10° sidewall tapering).^156–158^ Such channel geometries can lead to “shadowing” effects that, akin to the gas permeability, also disrupt the photopolymerization reactions required for DLW-based printing.^159–161^ In combination, these factors provide a basis for prior efforts in which microstructures were printed with attachments solely to the interior glass substrate of PDMS-on-glass microchannels, thereby bypassing the need for DLW in close proximity to the PDMS.^162–165^

**Fig. 5:**
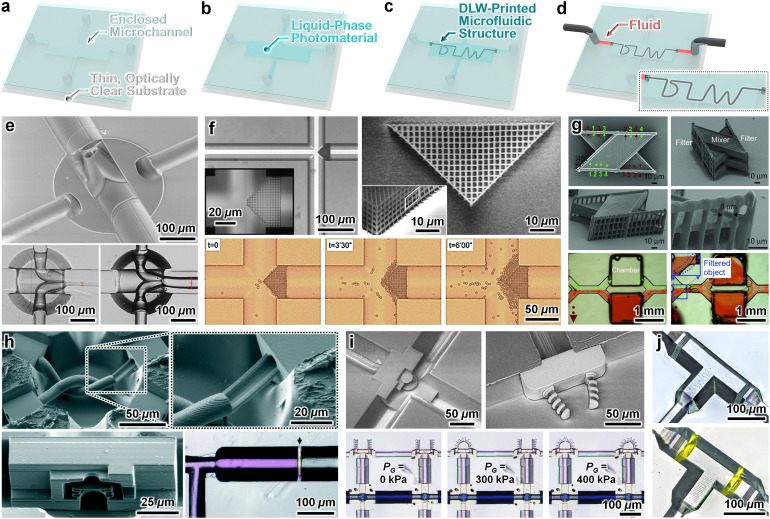
Microfluidic systems fabricated by DLW-printing 3D microstructures directly inside (and fluidically sealed to the entire luminal surfaces) of enclosed microchannels—strategies referred to as “*in situ* DLW (*is*DLW)”. (a–d) Conceptual illustrations of an example fabrication protocol. (a) Enclosed microfluidic device with tapered (≥30–34°) microchannel sidewalls and integrated macro-to-micro fluidic interfaces. (b) Liquid-phase photomaterial loaded into the device. (c) Microdevice after *is*DLW of “DLW” microfluidic structures. (d) Loading of fluid through the complete microfluidic system (*i.e.*, through the “DLW” microfluidic structures). (e–j) Representative examples in the literature. (e) A microfluidic spinneret *is*DLW-printed inside a PDMS-on-glass microfluidic system with sol–gel-coated microchannels.^169^ (f) A porous microfilter *is*DLW-printed inside a commercial borosilicate glass microchannel chip.^170^ (g) A micromixer with integrated filter structures *is*DLW-printed inside a glass microchip (produced by femtosecond laser-assisted wet etching).^175^ (h) Micrographs of (top) interweaving tubular microvessel structures, and (bottom) a microfluidic transistor (left) and fluidic barrier structure (right), which were all *is*DLW-printed inside microdevices composed of the thermoplastic material, cyclic olefin polymer (COP).^187^ (i) A microfluidic circuit comprising two sets of fluidic microgrippers and two distinct microfluidic transistors, which were all *is*DLW-printed inside of a COP–COP microdevice.^191^ (j) Micrographs (top) before, and (bottom) after *is*DLW-printing of microfluidic barrier structures inside of a COP–COP microdevice *via* a photografting approach (based on benzophenone (BP) surface modification).^192^

Many microfluidics applications, however, are founded on the ability to DLW-print microstructures that are fluidically sealed to the entire luminal surface of the microchannel. One route to bridge this gap is through the use of sealant glues. For example, Lölsberg *et al.* reported a strategy for DLW-printing a 3D microfluidic structure (a spider-inspired spinneret) inside of a PDMS-on-glass chip—comprising channels with 30° sidewall tapering to prevent shadowing effects—and then loading a silane-based epoxy *via* sacrificial side channels to limit fluidic leakage through any residual voids between the printed microstructure and the PDMS sidewalls.^166^ The majority of other efforts have instead focused on pathways that allow for microstructures to be DLW-printed directly onto (and fluidically sealed to) the entire interior surface (*i.e.*, all four walls) of the microfluidic channel—strategies termed “*in situ* DLW (*is*DLW)”. Such protocols involve four key steps. First, an enclosed microfluidic device—with macro-to-micro fluidic interfaces and tapered (≥30–34°) channel sidewalls—is fabricated (Fig. 5a). Second, photomaterial is loaded into the microchannel (Fig. 5b). Next, a microstructure is DLW-printed inside the microchannel, including regions attached to the entire luminal surface of the channel (Fig. 5c). Lastly, the prints are developed (*e.g.*, by perfusing developing agents and/or solvents through the microchannel to remove any residual uncured photomaterial), and the complete system can be employed for microfluidic applications (Fig. 5d).

For PDMS-on-glass microdevices, the primary means to enable *is*DLW of microfluidic structures involves performing surface modifications to the PDMS, namely, in the form of sol–gel coatings. For example, using an acid-catalysed sol–gel reaction to coat the PDMS surface with a siliceous layer of (3-aminopropyl)triethoxysilane (APTES) that inhibits oxygen permeability,^167^ Lamont *et al.* demonstrated *is*DLW-printed fluidically sealed microstructures in PDMS-on-glass channels up to 100 μm in height, with the caveat that the degree of sidewall tapering played a critical role in the efficacy of the structure-to-sidewall microfluidic sealing integrity.^124^ The Wessling group adapted this approach by instead using a photoreactive sol–gel coating process^168^ to facilitate *is*DLW-printing of microstructures including 3D microfluidic spinnerets for manufacturing wet-spun fibres for tissue engineering applications (Fig. 5e).^169^ The researchers reported that using the photoreactive sol–gel coating allowed the photoreactive moiety to bond with the photomaterial during the DLW process to produce a strong structure-to-channel adhesion, which they demonstrated by *is*DLW-printing a 10 μm-thick fluidic barrier (height ≈ 50 μm) that was able to withstand pressures of up to 300 kPa.^169^ There are two main drawbacks to these approaches. First, the protocols for performing the surface modifications to the PDMS-on-glass microchannels can be time and labour intensive and, based on the report by Lüken *et al.*, there can be challenges in lab-to-lab reproducibility (*e.g.*, for the acid-catalysed sol–gel reaction).^124,167,169^ In addition, the adhesion strength between the *is*DLW-printed structures and the inner surfaces appears to be limited—*i.e.*, in the range of 75 kPa (ref. 124) to 300 kPa (ref. 169)—which suggests that the target working pressure may be a determining factor as to whether or not such PDMS-based *is*DLW strategies represent a suitable option for a potential microfluidic application.

### 4.2. DLW inside enclosed glass microfluidic systems

The aforementioned challenges in facilitating direct DLW-printing of microstructures onto the entire luminal surface of microchannels for PDMS-on-glass devices have motivated researchers to investigate *is*DLW strategies for microfluidic systems based on alternative materials. Earlier such efforts focused on *is*DLW within glass microfluidic systems. For example, Amato *et al.* purchased a commercial cross-channel microfluidic chip made entirely of borosilicate glass and then *is*DLW-printed a 3D porous microfilter, which the researchers demonstrated for both filtration of 3 μm polystyrene spheres as well as plasma separation from whole blood (Fig. 5f).^170^ To bypass the need to purchase commercial chips while enabling higher customization of the bulk microfluidic system design, the Sugioka group harnessed their laser micromachining (*e.g.*, laser ablation) methods^171–173^ to create glass microdevices in which to *is*DLW-print microstructures.^174,175^ In particular, Wu *et al.* demonstrated microfilters capable of separating Euglena cells^174^ as well as a micromixer with integrated filtering structures for on-chip synthesis of ZnO particles (Fig. 5g).^175^ Both Kelemen *et al.* and Wang *et al.* extended this approach to print optical structures inside of glass chips, but these structures were not fluidically sealed to the microchannel lumens.^176,177^ The main drawbacks for such approaches stem from the deficits associated with glass microchip fabrication. For instance, the hybrid protocol described by Wu *et al.* relied on femtosecond laser microprocessing, HF wet etching, and multiple annealing steps to produce the glass microdevice.^175^ Many researchers in the *Lab on a Chip* community do not have the access and/or training required to perform such protocols, while those who do may still refrain from employing them due to concerns regarding safety (*e.g.*, for HF) as well as fabrication time, cost, and labour. Such barriers provide a possible basis for the lack of reports of fully glass microdevice-based *is*DLW in the literature in recent years.

### 4.3. DLW inside enclosed thermoplastic microfluidic systems

The thermoplastic material, cyclic olefin polymer (COP), has emerged as a promising alternative microdevice material for *is*DLW strategies due to several key benefits: (i) COP has high optical transparency;^178–180^ (ii) COP is resistant to polar organic solvents, such as those commonly used in DLW development protocols;^181,182^ (iii) COP exhibits low gas permeability;^183^ and (iv) COP supports accessible micropattern replication and bonding procedures akin to those used widely for PDMS.^184–186^ Alsharhan *et al.* first reported the ability to *is*DLW-print microfluidic structures directly onto (and fluidically sealed to) untreated microchannels of COP–COP microdevices.^187^ In addition to demonstrations of interweaving tubular microvessel structures (inner diameters <10 μm) and a normally open microfluidic transistor, the researchers observed that 10 μm-thick fluidic barriers with heights up to 100 μm were able to withstand pressures up to 500 kPa (*i.e.*, the limit of the experimental setup), with sidewall tapering again a key determinant in microfluidic sealing integrity (Fig. 5h).^187^ This COP-based *is*DLW strategy has since been extended to additional applications, including to produce retinal phantoms for advanced optics evaluations^188,189^ and to print 3D conductive microstructures within microfluidic channels.^190^ In the area of soft microrobotics, Alsharhan *et al. is*DLW-printed normally closed microfluidic transistors (with distinct activation pressures) as well as soft microgrippers in a COP–COP microchip, and demonstrated multiple actuation states by adjusting the magnitude of a single applied pressure (Fig. 5i).^191^

While these *is*DLW efforts that involved printing microstructures directly onto COP–COP microchannel surfaces without any modifications demonstrated markedly stronger structure-to-channel sealing compared to those of sol–gel-coated PDMS-on-glass systems,^124,169^ the maximum applied pressures investigated did not exceed 500 kPa.^187–191^ To provide a route to high-pressure microfluidic applications based on *is*DLW, Han *et al.* reported a photografting approaching to facilitate the formation of covalent bonds between the *is*DLW-printed microstructures and the COP–COP microchannels during the printing process.^192^ Notably, by incorporating an initial surface modification protocol in which a benzophenone (BP) solution (1.0 wt%) was loaded into the COP–COP microchannel and then irradiated in a UV chamber for 5 min (total dose ≈ 8 J cm^−2^), subsequent *is*DLW of 10 μm-thick fluidic barrier structures with heights of 60 μm withstood applied pressures of over 3 MPa, including cases as high as 7 MPa (Fig. 5j).^192^

### 4.4. “Oil-immersion” DLW configurations

**Fig. 6:**
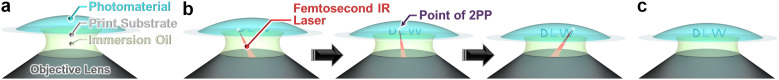
Conceptual illustrations of a representative “oil-immersion” configuration-based DLW manufacturing process to produce 3D “DLW” microstructures. (a) Uncured photomaterial atop a thin, optically transparent print substrate with immersion oil between the underside of the print substrate and the objective lens. (b) A pulsed IR laser is scanned through the immersion oil, print substrate, and then the photomaterial (including in some case, previously polymerized microstructures) to initiate 2PP in target locations. (c) Completion of the oil-immersion mode DLW printing process.

One of the most important caveats to these strategies that involve DLW-printing microstructures directly inside of fully enclosed microfluidic devices is that they rely on the use of “oil-immersion” DLW configurations,^124,162–166,169,170,174,175,187–191^ which have distinctive benefits and limitations. Oil-immersion DLW encompasses printing setups in which an immersion oil is applied between the objective lens and the bottom side of the optically transparent print substrate (Fig. 6a) to reduce refraction-related aberrations to the laser.^193–195^ Thus, the laser must pass through several different mediums to ultimately initiate photopolymerization at the voxel site: (i) the objective lens, (ii) the immersion oil, (iii) the substrate, and then (iv) the photomaterial of interest, which in some cases includes through previously polymerized microstructures (Fig. 6b). The primary advantage of oil-immersion configurations is the breadth of compatible photomaterials. Specifically, because the distance the laser passes through the photomaterial is relatively short compared to that of the immersion oil (and substrate), a wide range of photoresists and photocomposites can be printed *via* oil-immersion DLW.^196–200^

Unfortunately, the overall laser path for oil-immersion DLW configurations leads to several drawbacks. First, the print substrate must not only be optically transparent and sufficiently thin (*e.g.*, ≤170 μm), but also ideally have its refractive index match that of the immersion oil to limit diffraction.^14^ A second drawback stems from layer-by-layer inconsistencies in the laser path during the printing process. Specifically, as microstructures are printed at farther distances past the substrate (*e.g.*, to build taller structures), the laser path changes dynamically—*i.e.*, the distance through the immersion oil decreases as the substrate moves closer to the objective lens and the distance the laser travels through the photomaterial (and/or polymerized microstructures) increases (Fig. 6b). Consequently, effective oil-immersion DLW generally requires height-based compensations—*e.g.*, increasing the power and/or decreasing the scan speed of the laser with increasing height (*i.e.*, increasing distance past the substrate)—to ensure the laser exposure dosage is sufficient to successfully initiate photopolymerization at all target heights (Fig. 6c). Because such compensations are dependent on the substrate material and thickness, the optical properties of the photomaterial, and, in cases where the laser will need to pass through any cured photomaterial to reach taller locations, the specific architecture being printed, the necessary height-based laser parameter adjustments must typically be identified experimentally on a case-by-case basis. Elucidating these compensations can not only be time and labour intensive, but there are also practical limits to the tuneable laser parameters (*e.g.*, decreasing the scan speed too much will greatly increase print time and cost), which provides a basis for the scarcity of reports in the literature for oil-immersion DLW-printed structures taller than 100 μm.

For *is*DLW strategies in particular, the magnitude of laser parameter compensations can be mitigated in part by using a “ceiling-to-floor” DLW methodology in which structures are first printed at the top of the microchannel (*i.e.*, farthest away from the substrate) and then printed in a point-by-point, layer-by-layer process until completing the structure at the base substrate (*e.g.*, the glass substrate of a PDMS-on-glass microdevice or the thin COP film of a COP–COP microchip).^124,166,187,192^ Although such laser writing paths circumvent the need to compensate for the specific structure being printed (*i.e.*, as the laser does not pass through previously polymerized photomaterial in this case), height-based compensations for the substrate and photomaterial properties are still needed. For example, in the COP-based *is*DLW protocol reported by Alsharhan *et al.*, while the laser scanning speed was held constant at 10 mm s^−1^, the laser power had to be varied using an exponential relationship from 18 mW to 42 mW—corresponding to heights of 0 μm to 100 μm, respectively—to effectively resolve 100 μm-tall microstructures (comprising IP-L 780 photoresist), despite using a “ceiling-to-floor” printing routine.^187^ An additional caveat is that the aforementioned sidewall tapering requirement (*e.g.*, ≥30–34°) to prevent shadowing effects suggests that conventional microfabrication methods that produce 2.5D microchannels are not well suited for *is*DLW. Alternative mould fabrication approaches based on DLW (see section 2) that can facilitate quasi-2.5D moulds represent the most prominent route to address this issue at present; however, such methods lack the production capacity of established microfabrication protocols (*e.g.*, wafer-scale photolithography), thereby limiting their current utility primarily to research settings.

## 5. DLW of millimetre- and submillimetre-scale microfluidic components

### 5.1. DLW of microfluidic components for manual integration and/or assembly

**Fig. 7:**
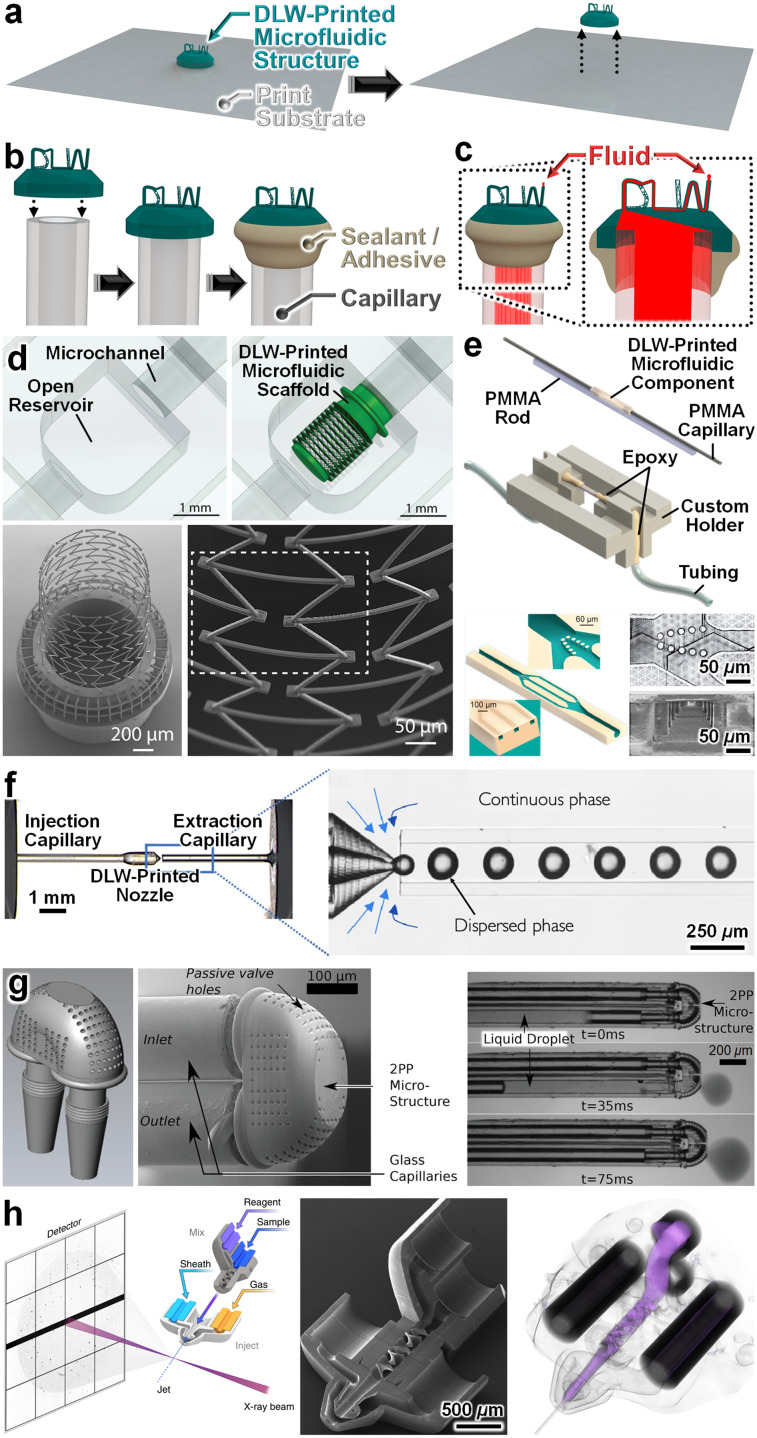
Microfluidic components fabricated by DLW-printing independent 3D microfluidic entities for subsequent manual fluidic interfacing. (a–c) Conceptual illustrations of an example fabrication protocol. (a) DLW-printing of an independent microfluidic entity and removal from the print substrate. (b) Manual interfacing of the DLW-printed microfluidic entity to a mesoscale fluidic capillary, followed by the application of a sealant/adhesive. (c) Loading of fluid into (and through) the complete microfluidic component. (d–h) Representative examples in the literature. (d) DLW-printed 3D cell scaffold manually interfaced with a fluidic channel.^203^ (e) DLW-printed nuclear magnetic resonance (NMR) microfluidic component manually interfaced with mesoscale fluidic capillaries with connections fluidically sealed using epoxy.^177^ (f) DLW-printed nozzle manually interfaced with (and glued to) a glass capillary for microdroplet generation.^207^ (g) DLW-printed microfluidic structure manually interfaced (without sealants/adhesives) with a capillary bundle for delivery and sampling of nanolitre volumes.^208^ (h) DLW-printed modular gas dynamic virtual nozzle (with integrated micromixers) manually interfaced with (and glued to) glass capillaries for serial femtosecond crystallography at X-ray free-electron lasers.^209^

Although DLW 3D printers lack the print fields and build volumes of their VPP counterparts,^201^ there are microfluidic applications for which it is advantageous to: (i) DLW-print 3D millimetre- or submillimetre-scale microfluidic components onto a sacrificial substrate (Fig. 7a – left), (ii) release the DLW-printed components from the substrate (Fig. 7a – right), and then (iii) manually interface macro-to-micro fluidic connections to the DLW-printed components (Fig. 7b) to facilitate microfluidic operations (Fig. 7c). For example, Michas *et al.* recently reported an approach for creating a “cardiac-pump-on-a-chip” system that involved DLW-printing an independent cellular scaffold microstructure and then manually inserting the component into the outlet port of a microchannel (Fig. 7d).^202^ To enable nuclear magnetic resonance (NMR) experiments on subnanolitre volumes, Montinaro *et al.* DLW-printed a microfluidic component with 3D microchannels that was manually interfaced with macroscale fluidic tubing *via* 200 μm-in-diameter intermediary capillaries that were sealed to the component's inlet and outlet ports as well as the tubing using epoxy (Fig. 7e).^203^ Similarly, researchers have manually sealed microfluidic components with a single input and/or output port to mesoscale fluidic tubing, syringe tips, and capillaries, such as microneedles,^204,205^ balloon catheters,^206^ 3D nozzles for droplet generation (Fig. 7f),^207^ and microfluidic delivery and sampling components (Fig. 7g).^208^ Notably, the Heymann and Chapman groups have used DLW to print and interface sophisticated 3D microfluidic nozzles, including those with integrated micromixers and up to four input ports (Fig. 7h),^209^ to facilitate biological imaging *via* X-ray free electron lasers.^209–211^

### 5.2. DLW of microfluidic entities directly atop meso/microscale fluidic components and systems

**Fig. 8:**
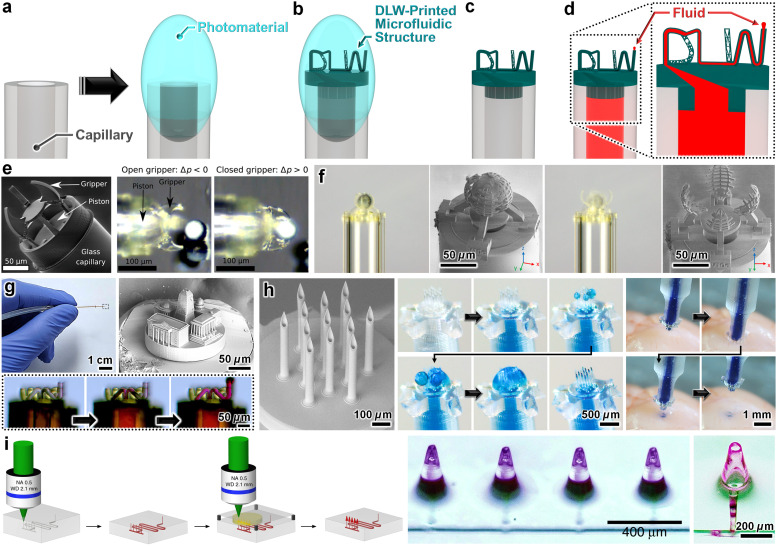
Microfluidic components fabricated by DLW-printing 3D microfluidic structures directly atop (and fluidically sealed to) meso/macroscale fluidic couplers and systems—strategies referred to as “*ex situ* DLW (*es*DLW)”. (a–d) Conceptual illustrations of an example fabrication protocol. (a) Photomaterial deposited on the tip of a mesoscale fluidic capillary. (b and c) “DLW” microfluidic structures *es*DLW-printed atop the capillary (b) before, and (c) after development. (d) Loading of fluid through the complete microfluidic component (*i.e.*, through and out of the “DLW” microfluidic structures). (e–i) Representative examples in the literature. (e and f) Micrographs of (e) micropiston-actuated microgrippers,^215^ and (f) pneumatically actuated bistable microgrippers,^216^*es*DLW-printed onto glass capillaries for manipulating microspheres.^215,216^ (g) Microfluidic structures with arbitrary geometries *es*DLW-printed onto fused silica glass capillary tubes and loaded with fluid.^217^ (h) Hollow microneedle arrays *es*DLW-printed onto “Digital Light Processing (DLP)” 3D-printed capillaries for injecting fluid into excised mouse brains.^218^ (i) Hollow conical microneedles *es*DLW-printed atop a microfluidic chip with external ports (prepared by femtosecond irradiation, annealing, grinding, and polishing).^220^

Outside of microfluidic fields, a common route to bypass the time and size restrictions of DLW is to print microstructures directly atop meso/macroscale components and devices, such as for optics,^212^ photonics,^213^ and microrobotics applications.^214^ Adapting such approaches for microfluidic scenarios, however, presents added challenges associated with facilitating the necessary fluidic pathways. Nonetheless, researchers have developed several hybrid strategies to DLW-print microfluidic entities directly onto (and fluidically sealed to) meso/macroscale fluidic components and systems. One prominent strategy—referred to as “*ex situ* DLW (*es*DLW)”—entails five main steps. First, photomaterial is placed at the tip of a meso/macroscale fluidic component with an externally accessible fluidic port, such as a capillary (Fig. 8a). Second, the component is loaded into the DLW 3D printer. Third, alignment protocols are performed with respect to the top surface of the fluidic component. Then, the microfluidic structure is DLW-printed directly atop the meso/macroscale component (Fig. 8b). Lastly, the assembly is removed from the printer to perform the development protocols (Fig. 8c), after which the complete system can be employed for microfluidic applications (Fig. 8d).

The Yang group has developed several such approaches to produce microrobotic components for medical applications, such as tissue biopsy and manipulation. In particular, Barbot *et al.* reported a method for DLW-printing a micropiston-actuated microgripper directly atop a 140 μm-in-diameter glass capillary, which they demonstrated by grasping, moving, and releasing 50 μm microspheres (Fig. 8e).^215^ In addition, Power *et al.* extended this approach with the addition of an oxygen plasma etching post-processing step to achieve a bistable, pneumatically actuated microgripper—DLW-printed onto a 170 μm-in-diameter hollow core, fused silica capillary tube with a polyimide coating—that was similarly capable of manipulating 50 μm microspheres (Fig. 8f).^216^

For applications that rely on the capacity to deliver fluids *via es*DLW-printed components, Acevedo *et al.* presented an approach for DLW-printing microfluidic structures with arbitrary geometries directly onto 360 μm-in-diameter fused silica tubes (Fig. 8g).^217^ Key drawbacks of the these approaches, however, stem from the need for custom-built capillary holders to support loading into the DLW 3D printer.^215–217^ Not only must each individual capillary be loaded into the holder manually (*i.e.*, by hand), but in cases with high numbers of capillaries, it can be difficult to maintain consistent surface heights and axial orientations for each capillary. As a result, the alignment protocols required for each individual capillary—which are typically performed manually (*i.e.*, by hand/eye under brightfield microscopy)—prior to initiation of the DLW printing process can be exceedingly time and labour intensive. To mitigate such issues, Sarker *et al.* reported a hybrid strategy that involved: (i) using VPP-based additive manufacturing (*e.g.*, digital light processing (DLP) 3D printing) to fabricate batch arrays of capillaries with tightly controlled positions and orientations, and then (ii) *es*DLW-printing 3D microfluidic structures—in this case, hollow microneedle arrays—directly atop each arrayed VPP-printed capillary.^218^ Using VPP to print the batch arrays of capillaries allowed the outer dimensions to be designed to support facile loading of the batch into the DLW 3D printer while also enabling customization of each arrayed capillary. The latter capability allowed the dimensions of the capillary base (*i.e.*, the end opposite that of the DLW-printed microstructure) to be designed to match those of the fluidic injector system, thereby obviating the need for additional fluidic couplers and/or sealants. The researchers demonstrated their *es*DLW-printed microneedle arrays by performing fluidic microinjection protocols with: (i) dyed aqueous fluids and suspensions of fluorescently labelled nanoparticles, which were each injected into excised mouse brains (Fig. 8h),^218^ and (ii) suspensions of dendritic cells and HEK293 cells.^219^

While these strategies involved DLW-printing onto mesoscale fluidic components with a single output port—namely, capillaries with outer diameters ranging from 140 μm to 1 mm—researchers have also reported the ability to DLW-print microfluidic structures directly atop bulk microfluidic systems with externally accessible fluidic ports. For example, Trautmann *et al.* demonstrated a hybrid femtosecond laser methodology that entailed: (i) a subtractive step in which 3D microchannels and external openings were produced for a polymethyl methacrylate (PMMA) microchip *via* femtosecond laser irradiation, annealing, grinding, and polishing, and then (ii) an additive step in which truncated cone-shaped hollow microneedles were DLW-printed directly atop each output opening (Fig. 7i).^220^ Similarly, Bohne *et al.* reported an approach for DLW-printing a 3D gas dynamic virtual nozzle directly onto the Si portion of a Si-glass microfluidic chip, with the print aligned to the through-holes (*i.e.*, outlet ports) of the Si to support the simultaneous loading of liquid water and He gas for the application of serial femtosecond crystallography.^221^ Recently, researchers have extended the approach by Sarker *et al.*^218^ to DLW-print 3D microfluidic structures directly atop VPP-printed 3D microfluidic devices, such as: (i) 3D coil structures that can be filled with liquid metal, such as eutectic gallium indium (eGaIn), to facilitate microelectronics applications,^222^ (ii) physiologically relevant PDMS-based 3D microvessels in which living cells can be seeded for “organ-on-a-chip” applications,^223^ and (iii) 3D coaxial micronozzles for microfluidic tubing fabrication.^224^

There are three primary considerations for employing these strategies for DLW-printing microfluidic structures atop such components and systems. First, the adhesion between the DLW-printed structures and the surface of the target fluidic components or systems must be sufficiently strong to prevent failures (*e.g.*, detachment of the DLW-printed structure under an applied pressure). A variety of factors can affect this adhesion strength, such as the preparation and roughness of the print surface as well as the material compatibility. Second, because the alignment protocols rely on optical detection of the print surface/location (*e.g.*, by eye under brightfield microscopy), the material of the target fluidic component or system must exhibit sufficient optical properties to support visibility. Lastly, the overall size of the target fluidic components and systems must be sufficiently small to be able to fit inside the DLW 3D printing system. For example, if glass capillary tubes are too long to be loaded into a target printer, they will need to be sectioned beforehand, which can necessitate couplers or adapters for ultimate use.

### 5.3. “Dip-in laser lithography (DiLL)” DLW configurations

A critical caveat to the aforementioned reports for DLW-printing millimetre- and submillimetre-scale microfluidic components is that the vast majority of these approaches rely on the use of “Dip-in Laser Lithography (DiLL)” DLW configurations.^202–211,215–219,221–223^ In contrast to oil-immersion DLW (Fig. 6), DiLL configurations involve immersing the objective lens directly in the photomaterial (Fig. 1e–g), which not only obviates the need for undesired height-specific laser dosage compensations, but also allows structures with heights in the millimetre-to-centimetre range to be printed. In addition, DiLL configurations circumvent the oil immersion-associated substrate constraints, allowing for DLW printing irrespective of the optical transparency and/or thickness of the substrate. The critical drawback of DiLL configurations stem from the material requirements for the photomaterial. Specifically, the photomaterial's refractive index (*n*) must match the specifications for a particular DLW 3D printer (*e.g.*, *n* ≈ 1.5 at 780 nm for Nanoscribe Photonic Professional systems) and—because the objective lens is fully immersed in the photomaterial (Fig. 1e–g)—it cannot contain any materials that could corrupt or degrade the lens (*e.g.*, certain solvents, acids, or bases).^14^ In combination, the range of photomaterials that are compatible with DiLL configurations is substantially smaller than that for oil-immersion DLW.

## 6. Future directions

**Fig. 9:**
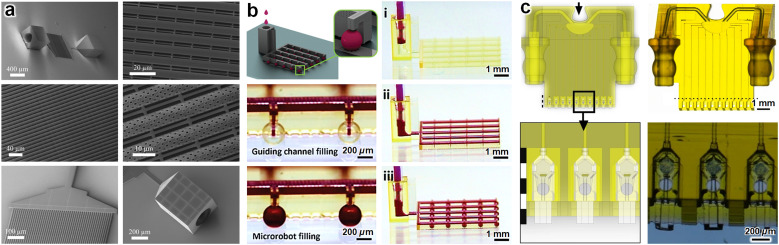
Fully DLW-printed microfluidic systems. (a) DLW-printed microfluidic system with one inlet and one outlet connected to 50 porous cylindrical microfluidic channels (with 1 μm array pores) for modelling the blood–brain barrier.^225^ (b) DLW-printed microfluidic system with one inlet for (i)–(iii) loading “microrobots” (*i.e.*, magnetic liquid–core–shell particles) for drug delivery applications.^226^ (c) DLW-printed microfluidic system with two inlets for culturing cell spheroids as well as mouse cumulus–oocyte-complexes and embryos.^227^

In contrast to the aforementioned hybrid approaches in which DLW is used in combination with additional fabrication methods, the “holy grail” for DLW-enabled microfluidic applications is the ability to print entire, centimetre-scale microfluidic systems without diminishing the feature resolution and overall production times of established DLW protocols. Recently, there have been a select number of efforts to use DLW to print millimetre- to centimetre-scale microfluidic systems. In particular, Marino *et al.* reported a DLW-printed blood–brain barrier model comprising one inlet and one outlet port connected to 50 cylindrical microfluidic channels (10 μm in diameter; 1 μm wall thickness) designed with 1 μm arrayed pores (Fig. 9a).^225^ At a larger scale, Sun *et al.* DLW-printed a microfluidic system for loading fluid into magnetic liquid–core–shell microparticles (referred to as “microrobots”) for drug delivery applications (Fig. 9b).^226^ The largest DLW-printed microfluidic system in the literature to date (8.8 mm × 8.2 mm × 3.6 mm) was reported by McLennan *et al.* for culturing cell spheroids as well as mouse cumulus–oocyte-complexes and embryos (Fig. 9c).^227^ This latter work is notable not only because—unlike the majority of works in the literature based on the use of commercial DLW printers from Nanoscribe GmbH—the authors used a NanoOne DLW printer from the company, UpNano GmbH, but also because the researchers leveraged a recent innovation for increasing the efficiency of DLW printing processes: “Dynamic Optical Tuning (DOT)”.^227,228^

**Fig. 10:**
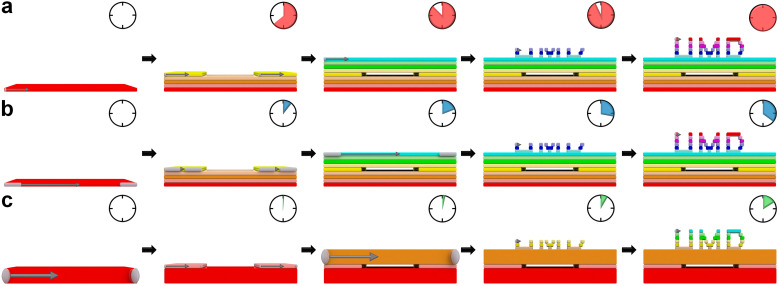
Conceptual illustrations of distinct DLW fabrication processes—based on using either a (a) fixed or (b and c) variable 2PP volume element (*i.e.*, “voxel”)—for a demonstrative “UMD” microstructure with an internal microchannel. (a) Fixed-voxel DLW. (b) “Adaptive resolution printing” DLW, which involves adjusting the voxel shape laterally, but not vertically, over the course of a DLW print run. (c) “Voxel tuning” DLW, which involves adjusting the voxel size dynamically (*i.e.*, scaled laterally and vertically) over the course of a DLW print run. Clocks denote example trends for elapsed print time corresponding to each process.

Historically, a key disadvantage of DLW has been that the size of the printing voxel remains fixed throughout the entire DLW printing process, which is inefficient for many scenarios that involve printing structures that include both: (i) regions with very fine details that require high feature resolution, and (ii) other regions with larger features that do not require such a high degree of precision. With a fixed voxel size, the smallest feature requirement dictates that designated size of the voxel, which is typically a function of the numerical aperture (NA) of the objective lens (*i.e.*, higher magnification objectives lead to smaller voxel sizes).^229–231^ As a result, even the bulkiest or coarsest parts of a structure will be printed with unnecessarily high resolution *via* relatively small voxels, thereby increasing the overall print time substantially (Fig. 10a). In addition, higher magnification objective lenses also lead to smaller print fields (*i.e.*, build areas), further diminishing production capabilities.

With DOT strategies such as “adaptive resolution printing” and “voxel tuning”, however, the size of the voxel can be adjusted dynamically during the DLW process—*e.g.*, to shrink or expand while printing fine or course features, respectively—to maximize print speed and efficiency (Fig. 10b and c).^232–234^ It is important to note that for voxel tuning approaches (Fig. 10c), while the height of the voxel size can be adjusted dynamically, the maximum print height (and overall build volume) of the DLW-printed structure remains dictated by the print volume of the specific printer being used. For example, the Nanoscribe Quantum X shape and the UpNano NanoOne 1000 have maximum print volumes of 50 × 50 × 12 mm^3^ and 100 × 120 × 40 mm^3^ (*L* × *W* × *H*), respectively, regardless of DOT implementation.

Nonetheless, should DOT strategies (*e.g.*, adaptive resolution printing and voxel tuning) become standard fixtures of DLW-based fabrication and enable the production time limitations associated with DLW-printing of entire millimetre- and/or centimetre-scale technologies to be addressed,^235,236^ it is possible that many of the hybrid DLW strategies highlighted in this *Tutorial Review* may no longer be needed for a myriad of microfluidic applications. With the recent emergence of commercial DLW printers equipped with such capabilities from companies such as Nanoscribe and UpNano, this scenario could soon come to fruition. Yet, to the authors' knowledge, only the aforementioned work by McLennan *et al.* has demonstrated the use of such DOT approaches for microfluidic applications;^227^ however, this lack of such reports is most likely a consequence of how recently such printers became available commercially (*i.e.*, within the past few years) and—due to their somewhat prohibitive costs (*e.g.*, typically >$600 000 USD)—the limited access microfluidics researchers have to such systems at present.

**Fig. 11:**
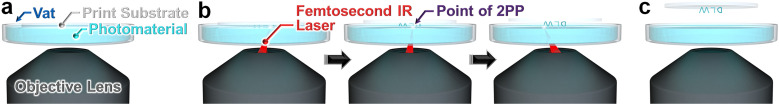
Conceptual illustrations of a representative “vat” configuration-based DLW manufacturing process to produce 3D “DLW” microstructures. (a) Uncured photomaterial inside a vat with a print substrate (in an inverted orientation) with its surface immersed in the photomaterial with an air objective lens positioned below the base of the vat. (b) A pulsed IR laser is scanned through the vat and then the photomaterial to initiate 2PP in target locations while the print substrate is raised up from the vat (layer by layer). (c) Completion of the vat DLW printing process.

An additional benefit of state-of-the-art printers such as those from UpNano is the use of “vat” DLW configurations, which offer a new avenue to combine the benefits of photomaterial selection associated with oil-immersion DLW configurations with the constant print parameters, substrate versatility, and print height advantages associated with DiLL DLW configurations. Vat DLW configurations resemble those of inverted VPP printers and include an open vat filled with photomaterial to allow for the print substrate to be introduced from above the vat while the objective lens (*e.g.*, an air objective) is positioned below the vat (Fig. 11a).^237^ During the DLW printing process, the print substrate is raised out of the vat while microstructures are photopolymerized underneath the substrate (Fig. 11b and c). In this configuration, the laser's path—*i.e.*, from the objective lens, through the vat, and then through the photomaterial—remains constant, thereby obviating the need for undesired height-based laser dosage compensations. Furthermore, because the photomaterial never interacts with the objective lens and the distance the laser passes through the photomaterial is relatively short, a broad range of photoresists and photocomposites can be printed using vat DLW—material selection that is further boosted by the high laser powers (*e.g.*, 1 W) of such systems. In combination with emerging DOT capabilities, the potential advent of practical one-step DLW-printing of entire microfluidic systems at size scales closer to those associated with conventional fabrication methods (*e.g.*, soft lithography), while still benefiting from the unparalleled geometric versatility, extensive photomaterial selection, and micron/submicron-scale feature resolutions afforded by DLW, holds distinctive promise to dramatically increase the adoption of DLW-based fabrication by the *Lab on a Chip* community.

## 7. Conclusions

Among the vast array of additive manufacturing technologies ranging from VPP and material jetting to extrusion-based methods, DLW offers a uniquely powerful pathway to enable fundamental research and translational applications that rely on both high geometric versatility and micron-to-submicron feature resolutions.^238–245^ For *Lab on a Chip* fields, however, this high resolution has represented a double-edged sword. Specifically, fabricating the larger-scale macro-to-micro fluidic interfaces (*e.g.*, input/output ports) that are essential to microfluidic systems *via* point-by-point scanning of a voxel with dimensions on the order of hundreds of nanometres to single-digit microns is inherently inefficient.^246,247^ As a result, researchers have devised a wide range of hybrid strategies to take advantage of DLW without undesired sacrifices in terms of manufacturing time, cost, and/or labour. Because each such strategy offers distinct capabilities and limitations, the choice of which should be employed for microfluidic system fabrication is often determined by application-specific requirements for designs, materials, and/or additional considerations.

For microfluidic applications that require quasi-2.5D microfluidic channels (*e.g.*, channels with many distinct heights and/or non-standard cross-sectional profiles), DLW can be used in place of conventional microfabrication processes such as photolithography for the fabrication of microchannel moulds and then integrated into standard microreplication protocols (*e.g.*, soft lithography). Alternatively, for cases that require microstructures (*e.g.*, with 2.5D, quasi-2.5D, or 3D geometries) inside of—but not fluidically sealed to—microfluidic channels, DLW can be used to print the structures in an unenclosed scenario, such as on a flat substrate or in an open microchannel, and then enclosed subsequently, such as with an open microchannel or a flat substrate, respectively. Although master mould fabrication and writing in an open channel are not unique to DLW-fabricated features and can be achieved by conventional cleanroom protocols, the primary benefit of using DLW is that it offers a far greater degree of customizability and 3D manufacturability that cannot be realized by standard microfabrication protocols. Historically, the drawback of using DLW in such scenarios has been throughput as DLW is not only much slower than photolithography, but also difficult to implement at similar wafer scales. It is important to note that recent developments for commercial DLW printers capable of two-photon grayscale lithography (*e.g.*, Nanoscribe “Quantum X” systems) and DOT (*e.g.*, Nanoscribe “Quantum X Shape” and UpNano “NanoOne” systems) have made considerable strides in bridging this gap to a degree, but matching the production speed and scale of photolithography (*e.g.*, rapid patterning of 4′′ and 6′′ wafers) appears to remain out of reach at present.

For microfluidic scenarios that demand and/or benefit from fully 3D micro/nanostructured technologies, we summarize general guidelines in Table 1 that can be applied on a case-by-case basis with regard to the specific requirements associated with a particular target application. For example, several *is*DLW strategies for printing microstructures inside of—and fluidically sealed to—enclosed microfluidic channels offer advantages in terms of material versatility, relatively rapid fabrication times (as DLW is only needed for the 3D structures of interest), and ease of incorporating high numbers of macro-to-micro fluidic interfaces. The limitations of *is*DLW mainly stem from those of oil-immersion configurations, including the need for: (i) a thin, optically transparent substrate (*e.g.*, glass or COP substrates with thicknesses ≤170 μm), (ii) undesired height-based laser dosage compensations that limit microstructure heights (*e.g.*, ≤100 μm), and (iii) microchannels to include tapered (*e.g.*, ≥30–34°) sidewalls to prevent shadowing effects that, in turn, necessitate non-standard channel microfabrication processes. It should be noted that previously reported VPP–DLW hybrid approaches^218,219,222–224^ could be adapted to facilitate *is*DLW directly inside of enclosed VPP-printed microfluidic devices; however, the VPP-printed microchips would need to satisfy all of the aforementioned *is*DLW criteria (Table 1). In contrast, DiLL-based DLW approaches—whether integration with meso/macroscale fluidic couplers is achieved through manual placement and assembly (*e.g.*, using sealant glues) or directly *via es*DLW strategies—offer benefits in terms of compatibility with print substrates irrespective of thickness or optical transparency, constant print parameters, and maximum print heights in the millimetre to centimetre range, but suffer from photomaterial constraints for optical properties (*e.g.*, refractive index, sufficient transparency) and constituents (*e.g.*, to prevent damage to the objective lens of the DLW printer). Emerging vat-based DLW configurations effectively resolve the deficits of DiLL configurations by facilitating vast material versatility while maintaining all of the advantages. Consequently, for printers capable of vat DLW, decisions between *es*DLW *versus* DLW-printing entire microfluidic technologies are essentially predicated on the materials, size, and production time requirements of an intended application (Table 1).

**Table 1:** Summary of key characteristics of primary DLW-based strategies for fabricating 3D microfluidic technologies. Green text = advantageous capabilities; red text = disadvantageous capabilities

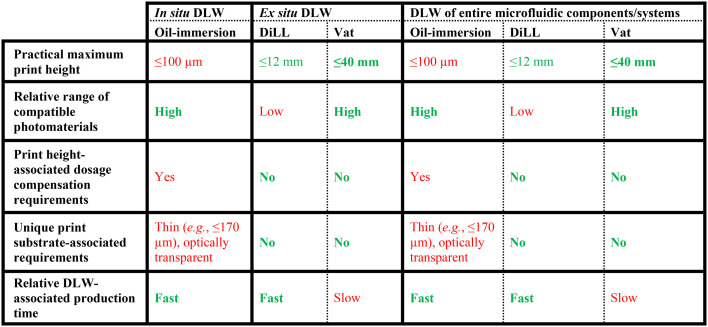

Although the major limitation of using DLW for printing millimetre- to centimetre-scale microfluidic components and systems has heretofore stemmed from the time (and cost) to print larger structures *via* inefficient point-by-point DLW with a micron- or submicron-scale voxel, importantly, the field is now at a turning point. Specifically, the recent release of commercial DLW printers equipped with DOT capabilities (*e.g.*, adaptive resolution printing and voxel tuning) that enable the voxel size and/or shape to be adjusted dynamically over the course of a DLW printing process (*i.e.*, to match the design resolution requirements) offers unprecedented potential for DLW to provide a practical and scalable means for 3D microfluidic system production. In the short term, DOT-enabled protocols could lead to the use of DLW for printing entire microfluidic components and systems becoming a suitable option for a far greater number of applications than ever before. In the long term, if these production speed and volume capabilities continue improving such that the gaps between DLW and larger-scale VPP 3D printing approaches can be effectively closed, it is possible that not only would many of the hybrid DLW strategies highlighted in this *Tutorial Review* become obsolete, but DLW could also emerge as the premier 3D printing technology for microfluidic system manufacturing.

## Conflicts of interest

There are no conflicts to declare.

## Supplementary Material
