# Correction: Direct laser writing-enabled 3D printing strategies for microfluidic applications

**DOI:** 10.1039/d4lc90040e

**Published:** 2024-04-22

**Authors:** Olivia M. Young, Xin Xu, Sunandita Sarker, Ryan D. Sochol

**Affiliations:** a Department of Mechanical Engineering, University of Maryland, College Park 2147 Glenn L. Martin Hall College Park MD 20742 USA rsochol@umd.edu; b Maryland Robotics Center, University of Maryland College Park MD 20742 USA; c Institute for Systems Research, University of Maryland College Park MD 20742 USA; d Fischell Department of Bioengineering, University of Maryland College Park MD 20742 USA; e Robert E. Fischell Institute for Biomedical Devices, University of Maryland College Park MD 20742 USA; f Department of Mechanical and Industrial Engineering, University of Massachusetts Amherst MA 01003 USA

## Abstract

Correction for ‘Direct laser writing-enabled 3D printing strategies for microfluidic applications’ by Olivia M. Young *et al.*, *Lab Chip*, 2024, DOI: https://doi.org/10.1039/D3LC00743J.

The authors regret that the published version of Table 1 contained incorrect descriptions in the final row. The correct descriptions are shown in the updated Table 1 here.

**Table 1:** Summary of key characteristics of primary DLW-based strategies for fabricating 3D microfluidic technologies. Green text = advantageous capabilities; red text = disadvantageous capabilities

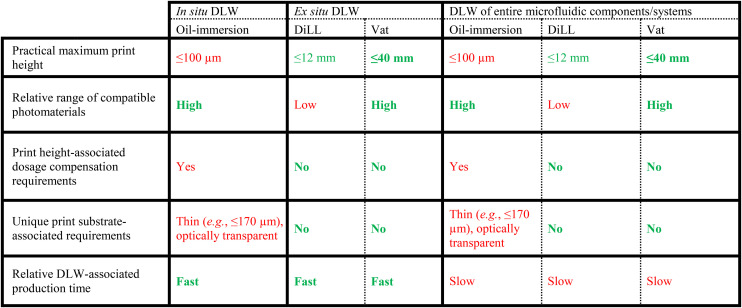

The Royal Society of Chemistry apologises for these errors and any consequent inconvenience to authors and readers.

## Supplementary Material

